# Beyond Suicidal Ideation: Identifying High-Risk University Students Through Depression, Sleep Disturbance, and Impulsivity—A Cross-Sectional Secondary Analysis

**DOI:** 10.3390/jcm15093236

**Published:** 2026-04-24

**Authors:** Valentina Baldini, Martina Gnazzo, Giorgia Varallo, Giuditta Bargiacchi, Ramona Di Stefano, Diana De Ronchi, Marco Carotenuto

**Affiliations:** 1 Department of Biomedical and Neuromotor Sciences, University of Bologna, 40126 Bologna, Italy; diana.deronchi@unibo.it; 2 Department of Biomedical, Metabolic and Neural Sciences, University of Modena and Reggio Emilia, 41125 Modena, Italy; giorgia.varallo@unimore.it; 3 Clinic of Child and Adolescent Neuropsychiatry, Department of Mental Health, Physical and Preventive Medicine, University of Campania “Luigi Vanvitelli”, 81100 Naples, Italy; martignazzo@hotmail.it (M.G.); giuditta.bargiacchi@studenti.unicampania.it (G.B.); marco.carotenuto@unicampania.it (M.C.); 4 Endocrinology and Medical Sexology (ENDOSEX), Department of Systems Medicine, Tor Vergata University of Rome, 00133 Rome, Italy; ramonadist@gmail.com

**Keywords:** suicidal ideation, depression, sleep quality, impulsivity, cannabis use, university students

## Abstract

**Background:** Suicide prevention strategies in university settings largely rely on detecting explicit suicidal ideation. However, students experiencing severe psychiatric distress may not endorse suicidal thoughts and therefore remain unidentified by ideation-centered screening models. This study aimed to identify and clinically characterize university students with high depressive symptoms, poor sleep quality, and elevated impulsivity who deny suicidal ideation in order to examine whether they represent a vulnerable yet overlooked subgroup. **Methods:** This cross-sectional secondary analysis included 814 undergraduate students from the National Sleep Research Resource (ANSWERS dataset). Participants were classified into three groups based on median splits of depressive symptoms (CES-D), sleep quality (PSQI), impulsivity (UPPS-P), and the presence or absence of suicidal ideation in the past three months: Invisible (high symptoms without ideation), Visible (high symptoms with ideation), and Healthy (low symptoms without ideation). Group differences were examined using ANOVA and chi-square tests. Multivariate logistic regression was conducted to assess independent predictors of suicidal ideation. **Results:** The Invisible group comprised 11.8% of the sample. Compared with Healthy participants, these individuals showed poorer sleep quality and higher levels of thwarted belongingness and perceived burdensomeness (all *p* < 0.001). Cannabis use was most prevalent in the Invisible group (54.2%), exceeding both Visible and Healthy groups (*p* < 0.001). In adjusted analyses, depressive symptoms (OR = 1.10, 95% CI: 1.08–1.12) and sleep disturbance (OR = 1.06, 95% CI: 1.01–1.12) independently predicted suicidal ideation, whereas impulsivity did not. **Conclusions:** A clinically meaningful subgroup of students experience severe psychological distress without endorsing suicidal ideation yet show behavioral and interpersonal vulnerability. These findings highlight a limitation of ideation-focused screening and support broader, symptom-informed mental health assessment strategies in university populations.

## 1. Introduction

Depression, sleep disturbances, and impulsivity rank among the most well-established transdiagnostic correlates of suicidal ideation and behavior [1,2,3]. In young adults and university populations—groups particularly vulnerable due to developmental transitions, academic stress, and identity formation—these symptoms are not only common but also frequently co-occur in ways that significantly impair functioning and increase psychiatric morbidity [4,5,6].

In recent years, there has been a marked increase in public and scientific attention toward mental health in young adults, particularly following the COVID-19 pandemic, which has been associated with a substantial rise in depressive symptoms, sleep disturbances, and suicidal behaviors in university populations [7,8]. At the same time, there has been growing recognition of the limitations of traditional suicide risk assessment models, especially those relying primarily on the explicit endorsement of suicidal ideation [9,10].

Consequently, suicide prevention efforts within these populations have primarily centered on identifying and treating individuals who report suicidal ideation, under the assumption that ideation is the most proximal and necessary antecedent of suicide attempts [11].

However, current screening methods in university settings mainly depend on explicitly asking about suicidal thoughts, even though increasing evidence shows that these methods might miss many at-risk individuals [9,10]. In modern clinical and public health approaches—especially those using digital and large-scale screening tools—this problem has become more significant, as relying on self-reported thoughts can underestimate risk in people who do not disclose or recognize suicidal feelings [12].

This may result in the systematic under-detection of a clinically relevant subgroup. While the presence of suicidal ideation is undeniably a strong predictor of suicide risk, its absence does not necessarily equate to safety, as previous studies have highlighted the limited predictive value of suicidal ideation alone in identifying individuals at risk of suicidal behavior [13]. The identification of psychiatric risk that is not accompanied by suicidal ideation—what might be described as “silent” or “invisible” risk—represents a critical gap in current clinical frameworks. In the present study, this concept is operationalized as the co-occurrence of elevated depressive symptoms, poor sleep quality, and increased impulsivity in the absence of self-reported suicidal ideation.

Importantly, this study does not assume that individuals in this group are necessarily at immediate risk of suicidal behavior. Rather, the aim is to identify a subgroup characterized by significant psychological distress and behavioral vulnerability, which may not be captured by ideation-based screening approaches. In this sense, the concept of “invisible” risk is intended to reflect a broader dimension of mental health burden that may, under certain conditions, confer increased vulnerability to suicidal outcomes.

The conceptual distinction between suicidal ideators and attempters has gained traction in the recent literature. Meta-analytic findings suggest that a significant proportion of suicide attempts occur in the absence of prior ideation, challenging linear models of suicide development [14]. Some theoretical models, including the Interpersonal Theory of Suicide and the Integrated Motivational–Volitional Model, acknowledge the role of cognitive, affective, and behavioral traits (e.g., impulsivity, burdensomeness, social disconnection) in facilitating suicidal behavior even without prolonged or clearly articulated ideation [15]. These frameworks suggest that risk is better understood as a dynamic and multidimensional process, one detectable through transdiagnostic markers rather than self-reported ideation alone.

Insomnia, for instance, has been shown to heighten emotional reactivity and reduce cognitive control, especially in individuals with comorbid mood symptoms [11]. Its presence is independently associated with an increased risk of suicide, even after controlling for depressive severity [12]. Likewise, impulsivity, particularly in its emotion-driven forms (positive and negative urgency), has been implicated in the transition from ideation to action [13]. In combination with depressive affect, these traits may act as accelerants for maladaptive coping strategies, substance use, and interpersonal dysfunction [14]. Yet clinical pathways often fail to consider the implications of these symptoms when suicidal ideation is absent. Importantly, these variables do not operate in isolation but may interact synergistically in increasing vulnerability to suicidal behavior. Depressive symptoms reflect affective distress and hopelessness, sleep disturbances contribute to emotional dysregulation and impaired cognitive control, while impulsivity—particularly urgency—facilitates the transition from distress to action.

Within this framework, the co-occurrence of these dimensions may represent a clinically meaningful risk profile, even in the absence of explicitly reported suicidal ideation.

Moreover, individuals with high levels of psychological symptoms but no suicidal ideation may exhibit behaviors or attitudes that obscure their risk profile. For example, alexithymia, emotional suppression, and fear of stigma may lead some individuals to underreport suicidal thoughts, despite experiencing overwhelming internal distress [15]. Others may be experiencing what has been described as non-suicidal distress, wherein the intensity of symptoms is not accompanied by a desire to die but is nonetheless associated with behavioral dysregulation, hopelessness, and interpersonal strain [16]. These individuals may be especially prone to engage in maladaptive behaviors such as substance use, particularly cannabis, as a means of affect regulation or social withdrawal [17].

Importantly, constructs such as perceived burdensomeness and thwarted belongingness—core dimensions of the Interpersonal Theory of Suicide—have been identified as key psychological mechanisms linking psychopathology and suicide risk [16]. While these constructs are typically studied in relation to suicidal ideation and behavior, their presence in individuals with a high symptom load but no ideation may indicate latent vulnerability. This vulnerability may not be immediately observable in standard clinical assessments but may nonetheless reflect an increased risk for future suicidal behavior under stress or acute crises. They may also serve as targets for early intervention in those who are emotionally dysregulated but not yet suicidal. From a theoretical perspective, these variables map onto core components of contemporary suicide models. Depressive symptoms are closely linked to feelings of hopelessness, sleep disturbances, impaired regulatory capacity, and impulsivity to the volitional phase of suicidal behavior. Together, they may reflect different stages of vulnerability within a transdiagnostic framework.

Despite the potential clinical importance of this population, relatively little attention has been paid to individuals characterized by a high transdiagnostic symptom burden in the absence of suicidal ideation. This gap is especially significant given recent efforts to adopt more dimensional and prevention-focused models of mental health, which highlight early identification of vulnerability beyond obvious suicidal thoughts [18].

To our knowledge, few studies have specifically focused on individuals characterized by a high transdiagnostic symptom burden in the absence of suicidal ideation, highlighting a gap in current research frameworks.

This group, which we term the “invisible”, is notably absent from most empirical models, which tend to dichotomize samples into suicidal vs. non-suicidal categories based solely on expressed ideation. As a result, much of the literature may underestimate the prevalence of high-risk profiles in young adults, particularly in university settings where symptom escalation can occur rapidly and where resources for mental health support are often constrained.

In this context, there is a growing need to refine suicide risk detection frameworks by incorporating broader symptom-based and transdiagnostic indicators. Such approaches may improve early identification of vulnerable individuals who remain undetected by ideation-centered models, particularly in high-risk populations such as university students.

The current study aims to address this gap by systematically identifying and characterizing the invisible group within a large sample of university students. Participants were classified into three groups based on their levels of depression, insomnia, and impulsivity, as well as the presence or absence of suicidal ideation: (I) invisible (high symptomatology without suicidal ideation), (II) visible (high symptomatology with suicidal ideation), and (III) healthy (low symptomatology, no ideation). We hypothesized that (1) the invisible group would differ significantly from healthy participants in terms of interpersonal risk factors (perceived burdensomeness and thwarted belongingness) and substance use (particularly cannabis); and (2) the invisible group would show a clinical profile partially overlapping with that of the visible group, despite the absence of suicidal ideation.

By illuminating the characteristics of this hidden subgroup, we aim to expand existing paradigms for suicide risk detection, challenge the adequacy of ideation-based screening, and emphasize the need for a more nuanced, symptom-informed approach to prevention in young adults.

## 2. Methods

### 2.1. Study Design and Participants

This study is a cross-sectional secondary analysis of the ANSWERS dataset, an epidemiological survey conducted between June 2020 and June 2021 among undergraduate students at a large U.S. university. The original study aimed to investigate sleep patterns and mental health outcomes, including depressive symptoms, sleep disturbances, and suicidal thoughts and behaviors. Data were collected via an anonymous web-based self-report survey, and no new data were collected for the present analysis. The dataset was obtained from the National Sleep Research Resource (NSRR) [19] and was previously analyzed in part by Tubbs and colleagues [20,21]. The primary aim of the original study was to evaluate various sleep parameters alongside mental health indicators, including suicidal thoughts and behaviors, with the aim of informing future longitudinal research in this population.

Participants were recruited through university communication channels and asked to complete an online survey. Responses were collected anonymously via a secure web platform. Participation was voluntary, and no direct incentives were offered.

The dataset includes demographic and self-reported questionnaire data from 971 young adult participants. The sample consisted of undergraduate students enrolled at a large U.S. university. Participants provided demographic information, including age and sex, along with self-reported measures of psychological and behavioral variables. The mean age of participants was approximately in the early twenties, consistent with a typical undergraduate population. For the present analysis, participants with missing data on sleep, depression, impulsivity, suicidal ideation, interpersonal risk factors, or substance use were excluded, resulting in a final sample of 814 participants.

Participants were categorized into three mutually exclusive groups based on symptom severity and suicidal ideation. Symptom severity was operationalized using median splits on the Center for Epidemiologic Studies Depression Scale (CES-D), the Pittsburgh Sleep Quality Index (PSQI), and the total score of the Short UPPS-P Impulsive Behavior Scale [21,22,23]. Participants scoring above the sample median on all three measures were considered to have high symptom severity. This approach was chosen to identify a clearly defined subgroup characterized by the co-occurrence of multiple high-risk dimensions, enhancing interpretability and clinical applicability.

Those with high symptom severity and no self-reported suicidal ideation in the past three months were classified as the invisible group. Those with similarly high symptom levels and reported suicidal ideation formed the visible group. Participants who scored at or below the sample median on all three symptom measures and denied suicidal ideation were categorized as the healthy group. Individuals with discordant symptom profiles (e.g., high depression but low impulsivity) or low symptoms combined with reported suicidal ideation were excluded from the comparative analyses.

Individuals with low symptom severity who reported suicidal ideation were excluded, as the study aimed to isolate the effects of high psychiatric burden in the presence or absence of suicidal thoughts. Sleep quality was assessed using the Pittsburgh Sleep Quality Index (PSQI), a widely used 19-item self-report instrument that evaluates subjective sleep quality and disturbances over the past month, with higher scores indicating poorer sleep quality [22]. Depressive symptoms were measured using the 20-item Center for Epidemiologic Studies Depression Scale (CES-D), which assesses the frequency of depressive symptoms in the general population, with higher scores reflecting greater symptom severity [23]. Impulsivity was evaluated using the Short UPPS-P Impulsive Behavior Scale, a validated measure that captures multiple dimensions of impulsivity, including negative urgency, positive urgency, a lack of premeditation, a lack of perseverance, and sensation seeking, with higher total scores indicating greater impulsivity [24].

Alcohol and cannabis use were assessed via dichotomous self-report items indicating lifetime use (ever vs. never; yes/no).

### 2.2. Ethics Statement

The original ANSWERS study received ethical approval from the relevant institutional review board, and all participants provided informed consent prior to participation. The dataset was fully anonymized before being made publicly available. The present study involved a secondary analysis of de-identified data and therefore did not require additional ethical approval.

### 2.3. Statistical Analysis

All statistical analyses were conducted using IBM SPSS Statistics, Version 29.0 (IBM Corp., Armonk, NY, USA). Participants were categorized into four mutually exclusive groups based on the severity of depressive symptoms, sleep disturbances, impulsivity, and the presence or absence of suicidal ideation: invisible (high symptoms without suicidal ideation), visible (high symptoms with suicidal ideation), healthy (low symptoms without suicidal ideation), and other (low symptoms with suicidal ideation). Analyses focused on comparing the invisible, visible, and healthy groups. Participants in the “other” group were excluded from group comparisons, as their symptom profile did not align with the study’s conceptual framework, which aimed to examine the role of high psychiatric burden in the presence or absence of suicidal ideation.

Continuous variables (e.g., PSQI, thwarted belongingness, perceived burdensomeness) were analyzed using one-way analysis of variance (ANOVA).

This approach was chosen to first explore between-group differences using univariate analyses (ANOVA and chi-square tests), followed by multivariate logistic regression to examine the independent contribution of each variable to suicidal ideation.

When significant main effects were detected, post hoc comparisons were performed using Bonferroni correction to account for multiple testing. Assumptions of normality and homogeneity of variance were examined prior to conducting ANOVA. Categorical variables (alcohol and cannabis use) were analyzed using Pearson’s chi-square test, and adjusted standardized residuals were inspected to identify cells contributing to significant associations.

In addition, a multivariate binary logistic regression analysis was conducted to examine the independent associations between depressive symptoms, sleep quality, impulsivity, and suicidal ideation. Suicidal ideation in the past three months (0 = absent, 1 = present) was entered as the dependent variable. Continuous total scores of depressive symptoms (CES-D), sleep quality (PSQI), and impulsivity (UPPS-P) were included as primary predictors. Prior to inclusion in the regression model, multicollinearity among predictors was assessed and found to be within acceptable limits.

Age, sex, cannabis use (yes/no), and alcohol use (yes/no) were entered as covariates to adjust for potential confounding effects. Results of the logistic regression analysis are reported as odds ratios (ORs) with 95% confidence intervals (CIs).

Statistical significance was set at *p* < 0.05 (two-tailed) for all analyses. The results are presented as means and standard deviations for continuous variables and as percentages for categorical variables.

## 3. Results

A total of 814 participants were included in the final analysis and classified into three groups based on symptom severity and suicidal ideation: invisible (*n* = 96; 11.8%), visible *(n* = 147; 18.1%), and healthy (*n* = 571; 70.1%). The mean age of the sample was 20.10 years (SD = 2.41), and 73.4% of participants were female. No significant differences in sex distribution were observed across groups.

The definitions of the groups are presented in Table 1.

Significant between-group differences emerged for all continuous clinical variables (Table 2). The invisible group reported significantly poorer sleep quality (PSQI: M = 9.27, SD = 3.65) than the Healthy group (M = 5.72, SD = 2.82; *p* < 0.001), but slightly better sleep than the Visible group (M = 10.07, SD = 3.53). Regarding social–interpersonal risk, the invisible group scored higher than the Healthy group on both thwarted belongingness (M = 31.23, SD = 11.08 vs. M = 21.84, SD = 10.40) and perceived burdensomeness (M = 9.62, SD = 5.25 vs. M = 7.19, SD = 3.13), though both measures remained significantly lower than in the Visible group (thwarted belongingness: M = 37.10, SD = 10.85; burdensomeness: M = 19.56, SD = 9.77; all *p* < 0.001).

Chi-square tests revealed no significant differences in alcohol use across groups (*p* = 0.24), with comparable prevalence between invisible (71.9%), visible (63.3%), and healthy (63.0%) participants. However, group differences in cannabis use were significant (*p* < 0.001). Cannabis use was reported by 54.2% of the invisible group, compared to 41.5% of the Visible group and 26.4% of the Healthy group.

To examine the independent associations between depressive symptoms, sleep quality, impulsivity, and suicidal ideation, a binary logistic regression analysis was conducted with suicidal ideation in the past three months (0 = absent, 1 = present) as the dependent variable. Continuous scores of depressive symptoms (CES-D), sleep quality (PSQI), and impulsivity (UPPS-P) were entered as primary predictors, adjusting for age, sex, cannabis use (yes/no), and alcohol use (yes/no).

To complement the bivariate analyses, a multivariate logistic regression model was conducted to examine the independent contribution of depressive symptoms, sleep quality, and impulsivity to suicidal ideation, adjusting for relevant covariates (age, sex, alcohol use, and cannabis use).

In the adjusted model, higher depressive symptom severity was significantly associated with increased odds of suicidal ideation (OR = 1.10, 95% CI: 1.08–1.12, *p* < 0.001). Poorer sleep quality also independently predicted suicidal ideation (OR = 1.06, 95% CI: 1.01–1.12, *p* = 0.028). In contrast, total impulsivity (UPPS-P) was not significantly associated with suicidal ideation after adjustment for covariates (OR = 1.01, 95% CI: 0.98–1.03, *p* = 0.697). None of the sociodemographic or substance use covariates reached statistical significance. The full results of the multivariate model are reported in Table 3.

**Figure 1:**
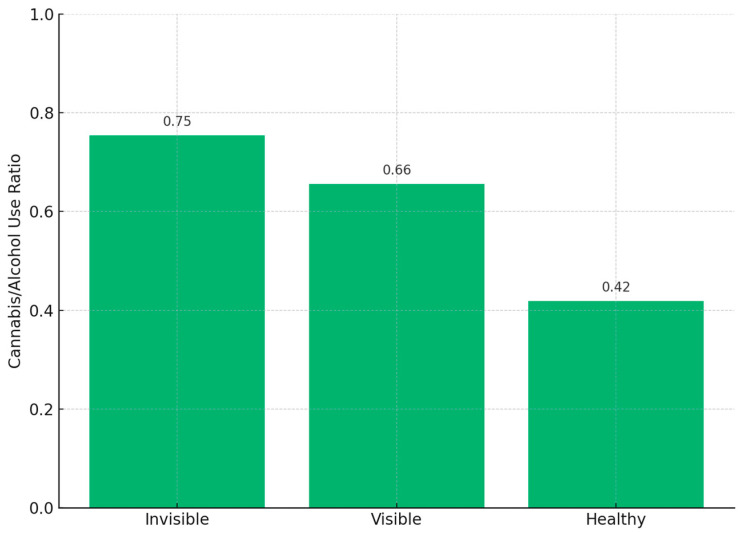
Ratio of cannabis to alcohol use by group. The Invisible group exhibited the highest proportion of cannabis use relative to alcohol use (0.75), compared to the Visible (0.66) and Healthy (0.42) groups.

To further investigate substance use profiles, we computed the ratio of cannabis to alcohol use for each group (Figure 1). The invisible group displayed the highest cannabis-to-alcohol ratio (0.75), indicating a stronger relative preference for cannabis compared to the visible (0.66) and Healthy (0.42) groups. This pattern highlights a potential behavioral distinction among students with high psychiatric symptoms in the absence of suicidal ideation.

## 4. Discussion

The key aims of this study were to identify and describe a subgroup of university students who show high levels of psychiatric symptoms but do not have suicidal thoughts and to compare them with both peers who have similar symptoms and have suicidal thoughts and healthy students. The study successfully found a distinct subgroup—called the invisible group—characterized by high depressive symptoms, poor sleep quality, and increased impulsivity, even though they do not have suicidal thoughts. Although these individuals did not express thoughts of death or self-harm, they showed significant psychological distress and behavioral risks.

Importantly, their profiles differed not only from those of healthy students with low symptom burden, but also from those of the visible group—students with similarly high symptomatology who did endorse suicidal ideation. These differences were especially noticeable in sleep quality, interpersonal distress, and cannabis use. The existence of this hidden subgroup highlights a significant limitation of traditional screening methods, which often rely solely on detecting suicidal ideation as a risk indicator [25,26]. These findings challenge the implicit assumption that the absence of suicidal ideation reflects low risk, suggesting instead that individuals may experience substantial psychological vulnerability even without endorsing suicidal thoughts. The findings suggest that a notable portion of high-risk individuals may go unnoticed when suicidal ideation is absent or unreported, emphasizing the need for more comprehensive, symptom-based approaches to suicide risk assessment. Importantly, the identification of this group should not be interpreted as indicating direct or imminent suicide risk. Rather, the “invisible” group appears to reflect a state of significant psychological distress and behavioral vulnerability that may remain undetected when screening relies exclusively on suicidal ideation. This distinction is crucial, as not all individuals with elevated psychiatric symptoms will develop suicidal behavior, but some may nonetheless represent a population requiring clinical attention and early intervention [27].

The invisible group showed sleep and affective impairments that were intermediate between the visible and healthy groups. Specifically, their mean PSQI score was substantially higher than that of the healthy group, indicating clinically poor sleep, but slightly lower than that of the visible group. This is consistent with the existing literature showing that sleep disturbances are a transdiagnostic vulnerability factor for emotional dysregulation, mood instability, and suicide risk, even when suicidal ideation is not present [10,25]. Likewise, levels of thwarted belongingness and perceived burdensomeness were elevated in the invisible group, reflecting significant interpersonal strain. Although these constructs are classically studied in relation to suicidal ideation and behavior [24,28], our findings suggest that they are also relevant for understanding psychological suffering in individuals who do not express suicidal thoughts.

A further mechanism that may help explain the clinical vulnerability of the “invisible” group concerns the effects of sleep deprivation on frontolimbic connectivity and behavioral regulation. Experimental and neuroimaging studies consistently show that sleep loss is associated with functional disconnection between the amygdala and prefrontal control regions, particularly the medial and dorsolateral prefrontal cortex. Under sleep deprivation, the amygdala exhibits exaggerated reactivity to emotional stimuli, while top-down inhibitory control from the prefrontal cortex is markedly reduced [29,30]. This neurobiological pattern mirrors that observed in individuals characterized by affective instability, impulsivity, and impaired decision-making.

Beyond emotional dysregulation, sleep deprivation has been shown to alter higher-order cognitive and moral processes. Laboratory studies indicate that insufficient sleep increases risk-taking, reduces sensitivity to negative consequences, and alters ethical and moral decision-making, leading to more self-interested, norm-violating, or dishonest behavior [31,32]. These effects appear to be mediated by impaired prefrontal functioning and reduced integration of affective signals into decision-making.

Within this framework, individuals in the invisible group, who report poor sleep quality yet deny suicidal ideation, may still experience heightened emotional reactivity alongside weakened cognitive and behavioral control. This condition may not present as explicit suicidal thoughts but rather as behavioral dysregulation, substance use, and interpersonal difficulties, thereby increasing vulnerability to impulsive or unplanned self-harm under acute stress. From a clinical perspective, this supports the view that sleep disturbances may act not only as correlates of suicidal ideation but also as transdiagnostic facilitators of risk by disrupting the neural systems underlying emotional regulation, moral judgment, and behavioral inhibition.

One of the most salient findings was the high prevalence of cannabis use in the invisible group. Over half of these individuals reported lifetime use of cannabis—substantially more than in the Visible group and more than double the rate observed in the Healthy group. Interestingly, the invisible group also exhibited the highest cannabis-to-alcohol use ratio, suggesting a relative preference for cannabis as a coping mechanism or form of self-regulation. This pattern may reflect attempts to manage dysphoric affect, anxiety, sleep difficulties, or social disconnection through substance use, particularly among individuals who are reluctant to disclose or confront suicidal ideation. Prior research has shown that cannabis use is often employed by young adults as a means of affect modulation and stress management, particularly in the context of psychiatric vulnerability [33,34,35].

However, the relationship between cannabis use, sleep disturbance, and mood symptoms is likely bidirectional. Cannabis may be used as a maladaptive coping strategy to manage sleep difficulties or emotional distress, while at the same time potentially exacerbating sleep disruption, affective instability, and cognitive dysregulation.

Indeed, this behavior may mask underlying risk by providing transient relief while maintaining emotional suppression and social disengagement.

The concept of the “invisible” group also aligns with emerging theoretical critiques of ideation-focused suicide models. While many frameworks, including the Interpersonal Theory of Suicide [36] and the Integrated Motivational–Volitional Model [37], recognize that suicidal ideation is not always a precursor to attempts, clinical practice often continues to rely on the presence of explicit suicidal thoughts as a gatekeeper for intervention. However, longitudinal studies and meta-analyses have shown that a significant proportion of suicide attempts occur in individuals who did not previously endorse ideation [7,38]. Our data underscore this concern: the invisible group exhibits traits commonly linked to suicide risk, such as impulsivity, poor sleep, substance use, and interpersonal distress, despite the absence of ideation. These individuals may represent a group at elevated latent risk who could transition rapidly to suicidal behavior under acute stress or crisis.

Clinically, this study highlights a critical blind spot in mental health screening practices. University campuses increasingly employ brief risk assessments and digital tools to identify students in crisis [39,40]. In practical terms, screening protocols based solely on suicidal ideation may fail to identify a subgroup of students who are already experiencing clinically significant distress but remain undetected within existing systems.

While these are valuable, they often prioritize detecting suicidal ideation as the primary indicator of need. Our findings suggest that such strategies may fail to capture a sizable segment of students who, while not endorsing suicidal thoughts, still experience serious psychological difficulties and engage in potentially harmful behaviors. The current findings advocate integrating broader symptom-based criteria, particularly those involving sleep, impulsivity, and social disconnection, into risk screening protocols. Doing so may help identify students in the early stages of distress who would otherwise remain invisible to the clinical system until their difficulties escalate.

An additional aspect that warrants consideration is the potential role of sex and gender differences in shaping the observed patterns. Although no significant differences in sex distribution were found across groups, it is well established that males and females may differ in the expression of depressive symptoms, sleep disturbances, impulsivity, and substance use behaviors [41,42]. For instance, females tend to report higher levels of internalizing symptoms such as depression and sleep difficulties, whereas males may exhibit higher levels of externalizing behaviors, including substance use and impulsivity.

These differences may influence not only the manifestation of psychological distress but also help-seeking behaviors and the likelihood of reporting suicidal ideation. In particular, some individuals—especially males—may be less likely to disclose suicidal thoughts despite experiencing significant distress, potentially contributing to the identification of “invisible” high-risk profiles [42,43].

From a clinical perspective, these findings highlight the importance of adopting gender-sensitive screening and intervention strategies, which take into account different pathways to distress and risk. Future research should further explore sex- and gender-specific mechanisms using stratified analyses or interaction models to better understand how vulnerability unfolds across different populations.

From a clinical and service perspective, these findings indicate that university mental health services could benefit from adding brief symptom-based screening tools into existing prevention strategies. In practice, this might involve using standardized measures of sleep quality (e.g., PSQI), depressive symptoms (e.g., CES-D), and impulsivity in routine digital or face-to-face screening procedures, even if students do not report suicidal thoughts. Students showing high symptom levels across multiple areas could then be provided with early, easily accessible interventions, such as psychoeducation, sleep-focused programs, or short-term psychological support. Furthermore, stepped-care models could be adjusted to treat this group as a priority for monitoring, rather than relying only on triage based on ideation. These approaches may enhance early detection and lower the risk of escalation for individuals who might otherwise go unnoticed.

Furthermore, the high rates of cannabis use among invisible participants raise important questions for prevention and health education. While cannabis is increasingly perceived as a benign or normalized substance among young adults, its use in the context of poor sleep, mood dysregulation, and social withdrawal may represent a maladaptive trajectory. These findings suggest the need for targeted interventions that address not only substance use per se, but the emotional and social contexts that make it appealing to at-risk individuals. Interventions targeting emotion regulation, peer connectedness, and non-substance-based coping strategies could be particularly beneficial in this subgroup.

This study is not without limitations. First, the cross-sectional design prevents any conclusions regarding directionality or causality among the observed relationships. It remains unclear whether poor sleep quality constitutes a risk factor for suicidal ideation or a consequence of it, or whether impulsive behavior precedes or results from substance use. Future longitudinal or prospective studies are needed to clarify the temporal sequence of these phenomena and to identify potential causal pathways linking them.

A further limitation concerns the assessment of suicidal ideation, which was based on a single dichotomous self-report item (“yes/no in the past three months”). While this approach is practical for large-scale epidemiological surveys and has been widely adopted in population-based research, it does not capture important clinical distinctions such as passive versus active ideation, intensity, persistence, or degree of impulse control. In adult populations, suicidal ideation represents a heterogeneous and clinically meaningful construct, and its presence is generally considered a psychiatric emergency regardless of severity. The use of a binary item, therefore, limits the ability to detect subthreshold, fluctuating, or ambivalent forms of ideation and may result in an underestimation of the true prevalence and complexity of suicidal thoughts. Future research should employ multi-item, validated instruments—such as the Beck Scale for Suicidal Ideation or the Columbia–Suicide Severity Rating Scale—to provide a more nuanced and clinically informative assessment of suicide risk.

The grouping strategy, based on a simultaneous median split of depressive symptoms (CES-D), sleep quality (PSQI), and impulsivity (UPPS-P), represents another methodological limitation. Although this approach allowed for the identification of a clearly defined subgroup characterized by a convergent and high psychiatric burden, it is inherently arbitrary and reduces the dimensional complexity of the data. In particular, requiring participants to exceed the median on all three measures may have excluded clinically relevant profiles, such as individuals with severe insomnia and depressive symptoms but only moderate impulsivity. This criterion increases group selectivity while reducing representativeness and may underestimate the heterogeneity of high-risk presentations.

This selection strategy may also limit external validity, as individuals with partially overlapping or discordant symptom profiles were excluded, potentially reducing the representativeness of the analyzed sample.

Importantly, the absence of data on suicidal behaviors (e.g., suicide attempts or non-suicidal self-injury) represents a key limitation. As a result, it is not possible to determine whether individuals in the “invisible” group are at increased risk of engaging in suicidal behavior. Therefore, the findings should be interpreted as reflecting psychological vulnerability rather than direct suicide risk. Future studies should incorporate detailed assessments of suicidal behaviors to better clarify the clinical significance of this subgroup within a suicide prevention framework.

Future studies should therefore favor dimensional or data-driven approaches, including multivariate regression models, clustering techniques, or latent class analyses, to better capture the continuity and complexity of psychiatric vulnerability.

The measurement of substance use also poses interpretative challenges. The dichotomous “ever used” classification does not distinguish between occasional, habitual, or problematic use, nor does it capture motivational aspects such as self-medication versus social or recreational use.

In addition, the dataset did not include information on other substances or psychotropic medications (e.g., benzodiazepines), which may also influence sleep, mood, and behavioral regulation.

Consequently, associations between substance use and suicidal ideation should be interpreted as preliminary. Future studies should incorporate more detailed quantitative and qualitative assessments to better characterize substance use patterns and their clinical significance.

From a statistical perspective, the reliance on bivariate analyses (ANOVA and chi-square tests) limits the ability to account for potential confounders. More sophisticated multivariate models—such as logistic or linear regression analyses—would allow for the inclusion of relevant covariates (e.g., age, sex, socioeconomic status, psychiatric history), thereby clarifying the independent contribution of each psychological factor.

In particular, relevant confounding variables such as psychiatric history, psychotropic medication use, socioeconomic factors, and recent life stressors were not available in the dataset and could not be included in the analyses, potentially influencing the observed associations.

In addition, the absence of sensitivity analyses and limited information regarding missing data management may affect the robustness and generalizability of the findings. Future research should adopt strategies such as multiple imputation and sensitivity testing to minimize potential bias.

Interpretatively, the observed associations suggest a possible interaction between depressive symptoms, sleep disturbances, and impulsive traits in conferring vulnerability to suicidal ideation. However, the modest effect sizes indicate that these factors alone are unlikely to fully explain suicide risk. Other dimensions—such as emotion regulation capacities, social connectedness, and coping strategies—may act as mediators or moderators. Future research should therefore investigate integrated, multifactorial, and transdiagnostic models combining psychological, biological, and contextual variables.

Alternative analytical approaches, such as decision tree models or machine learning techniques, may offer additional insights into complex interactions among risk factors and could enhance the identification of high-risk subgroups in future studies.

Finally, the generalizability of the present findings is limited by the use of a single university-based sample, which may not be representative of the broader population of young adults or individuals outside academic settings. Replication in multicenter, cross-cultural, and age-diverse cohorts will be essential to validate these preliminary observations and to inform more inclusive and generalizable prevention strategies.

In conclusion, this study provides a valuable initial insight into subtle psychological vulnerabilities that may underlie suicidal ideation among ostensibly healthy young adults. However, its findings should be viewed as exploratory rather than definitive. The main takeaway is that depressive symptoms, poor sleep, and impulsivity represent potential indicators of latent risk rather than deterministic predictors. Clarifying causal mechanisms, testing mediation and moderation effects, and adopting robust multivariate models in longitudinal frameworks represent key directions for future research aimed at developing reliable screening and early prevention strategies.

Despite these limitations, this study offers a novel contribution by empirically identifying a subgroup of high-symptom individuals who remain undetected by ideation-focused screening methods. The findings support a reconceptualization of suicide prevention approaches that extend beyond self-reported suicidal thoughts, emphasizing instead broader symptom-informed frameworks capable of capturing risk in its early, less visible forms.

## 5. Conclusions

In conclusion, students who experience severe emotional distress but do not report suicidal ideation should not be assumed to be low-risk. Their clinical profiles—marked by poor sleep, interpersonal strain, impulsivity, and cannabis use—indicate a need for early recognition and intervention. These individuals are not merely “subclinical” or “resilient,” but may in fact be silently struggling with psychological burdens that current systems fail to address. Expanding our detection strategies to identify such invisible distress could represent a critical step in improving mental health care and suicide prevention in young adult populations.

These findings highlight the need to reconceptualize suicide prevention not solely as a response to suicidal ideation but as a broader effort to detect psychiatric risk through behavioral and interpersonal indicators. Recognizing and supporting individuals with “silent suffering” is not only a clinical priority but also a matter of equity in access to care.

## Figures and Tables

**Table 1:** Participant group definitions.

Group	Definition	n (%)
**Invisible**	High levels of depression, insomnia, and impulsivity, but no suicidal thoughts	96 (11.8%)
**Visible**	High levels of depression, insomnia, and impulsivity, with suicidal thoughts	147 (18.1%)
**Healthy**	Low levels of depression, insomnia, and impulsivity, and no suicidal thoughts	571 (70.1%)

Groups were defined based on symptom levels (depression, insomnia, impulsivity) and the presence of suicidal ideation.

**Table 2:** Clinical and behavioral characteristics by group.

Variable	Invisible (n = 96)	Visible (n = 147)	Healthy (n = 571)	*p*-Value
PSQI, mean (SD)	9.27 (3.65)	10.07 (3.53)	5.72 (2.82)	<0.001
Thwarted belongingness, mean (SD)	31.23 (11.08)	37.10 (10.85)	21.84 (10.40)	<0.001
Perceived burdensomeness, mean (SD)	9.62 (5.25)	19.56 (9.77)	7.19 (3.13)	<0.001
Alcohol use, n (%)	69 (71.9%)	93 (63.3%)	360 (63.0%)	0.24
Cannabis use, n (%)	52 (54.2%)	61 (41.5%)	151 (26.4%)	<0.001

Values are presented as mean (SD) for continuous variables and n (%) for categorical variables. PSQI = Pittsburgh Sleep Quality Index.

**Table 3:** Multivariate logistic regression analysis predicting suicidal ideation.

Predictor	OR	95% CI	*p* Value
CES-D	1.103	1.082–1.124	<0.001
PSQI	1.062	1.006–1.120	0.028
UPPS-P	1.005	0.980–1.031	0.697
Age (years)	0.966	0.916–1.018	0.195
Male Sex	0.790	0.511–1.221	0.288
Cannabis use	1.091	0.723–1.646	0.680
Alcohol use	1.246	0.818–1.897	0.305

CES-D = Center for Epidemiologic Studies Depression Scale; PSQI = Pittsburgh Quality Index; UPPS-P = Impulsive Behavior Scale. Model χ^2^ = 152.84, *p* < 0.001; Nagelkerke R^2^ = 0.29; Intercept (constant) OR = 0.42, *p* = 0.018.

## Data Availability

The data used in this study are publicly available through the National Sleep Research Resource (NSRR) repository. Access to the ANSWERS dataset can be requested at https://sleepdata.org (accessed on 15 September 2025).
